# Advanced Etching Techniques of LiNbO_3_ Nanodevices

**DOI:** 10.3390/nano13202789

**Published:** 2023-10-18

**Authors:** Bowen Shen, Di Hu, Cuihua Dai, Xiaoyang Yu, Xiaojun Tan, Jie Sun, Jun Jiang, Anquan Jiang

**Affiliations:** School of Microelectronics, Fudan University, Shanghai 200433, China; 20112020023@fudan.edu.cn (B.S.); 21112020010@m.fudan.edu.cn (D.H.); 21112020078@m.fudan.edu.cn (C.D.); 21112020033@m.fudan.edu.cn (X.Y.); 20112020031@fudan.edu.cn (X.T.); jie_sun@fudan.edu.cn (J.S.)

**Keywords:** ferroelectric memory, LiNbO_3_, dry etching, wet etching, proton exchange

## Abstract

Single LiNbO_3_ (LNO) crystals are widely utilized in surface acoustic wave devices, optoelectronic devices, and novel ferroelectric memory devices due to their remarkable electro-optic and piezoelectric properties, and high saturation and remnant polarizations. However, challenges remain regarding their nanofabrication that hinder their applications. The prevailing etching techniques for LNO encompass dry etching, wet etching, and focused-ion-beam etching, each having distinct merits and demerits. Achieving higher etching rates and improved sidewall angles presents a challenge in LNO nanofabrication. Building upon the current etching researches, this study explores various etching methods using instruments capable of generating diverse plasma densities, such as dry etching in reactive ion etching (RIE) and inductively coupled plasma (ICP), proton exchange-enhanced etching, and wet chemical etching following high-temperature reduction treatment, as well as hybrid dry and wet etching. Ultimately, after employing RIE dry etching combined with wet etching, following a high-temperature reduction treatment, an etching rate of 10 nm/min and pretty 90° sidewall angles were achieved. Furthermore, high etching rates of 79 nm/min with steep sidewall angles of 83° were obtained using ICP dry etching. Additionally, using SiO_2_ masks, a high etching rate of 108 nm/min and an etching selectivity ratio of 0.86:1 were achieved. Distinct etching conditions yielded diverse yet exceptional results, providing multiple processing paths of etching for the versatile application of LNO.

## 1. Introduction

LNO, an artificially grown single crystal, boasts rich and extensive researches in the history. It possesses a unique combination of exceptional properties, including piezoelectricity, the electro-optic effect, acousto-optic effect, nonlinear optical behavior, and optical birefringence [1,2,3,4,5]. With its impressively high electro-optic coefficient, high intrinsic bandwidth, elevated refractive index, and wide transparency range in the light frequency, LNO has emerged as a remarkably versatile and captivating photonic material [6,7,8,9]. Moreover, its substantial electromechanical coupling coefficient and low propagation loss have positioned it as a focal point for research in surface acoustic wave devices [10,11]. In recent years, the exploration of domain-wall conductivity has triggered a surge in the development of domain wall devices [12,13,14,15,16,17]. LNO, characterized by its high Curie temperature, large saturation polarization and remanent polarization, has taken center stage in the realm of domain wall memory-device research [18,19,20,21,22,23,24,25]. Despite its immense potential, LNO still encounters certain challenges in practical applications. It currently lags behind its integrated photonics counterparts in optoelectronic applications, and the commercialization of domain wall memory chips, centered around LNO, has yet to be realized. The primary impediment lies in the formidable processing difficulties associated with LNO materials [26,27,28,29].

The nanofabrication processes of LNO mainly involve several techniques, such as dry etching, wet etching, and focused-ion-beam etching [30,31,32]. Dry etching can be further divided into F-based-gas and Cl-based-gas dry etching. With the dry-etching process with F-based gas, LiF by-products are generated. Due to its high melting point, LiF is not easily removed and tends to accumulate on the sidewalls, resulting in a reduced etching sidewall angle. For instance, in 2023, Kozlov et al. used SF_6_ and Ar in ICP to fabricate LNO optical waveguide devices, but the sidewall angle of the waveguide was only 60–75° [33]. On the other hand, Cl-based gas generates LiCl by-products during the etching of LNO, and as LiCl has a lower melting point, it can be easily removed without accumulating on the sidewalls, ultimately leading to better etching morphology. Additionally, Cl-based gas avoids the consumption of non-metal masks, addressing the compatibility issues between metal masks and traditional semiconductor processes. However, the etching rate of Cl-based gas is not as high as F-based gas, and it demands strict requirements for etching equipment, limiting its use in large-scale production and deep-etching processes. For instance, in 2019, Bahadori et al. utilized a SiO_2_ mask and a mixture of Ar, Cl_2_, and BCl_3_ in ICP to achieve an etching selectivity ratio of 1.45:1, and the sidewall angle reached 83°, presenting a smooth LNO etching surface [34]. Similarly, in 2020, Shen et al. employed a SiO_2_ mask and a mixture of Ar, Cl_2_, and BCl_3_ as the etching gas, obtaining an etching sidewall angle close to 80° [35].

Wet etching includes acid wet etching (HF/HNO_3_) and alkaline wet etching (H_2_O_2_/NH_4_0H/H_2_O (SC-1) solution, NaOH solution) [10,36]. The wet-etching process is isotropic and results in a lower sidewall angle during the etching of LNO. Additionally, several studies have demonstrated that the wet etching of LNO can lead to non-uniform etching morphology due to different etching rates along different crystal orientations. For instance, in 2022, Zhuang et al. used SC-1 solution to etch LNO samples covered with a SiO_2_ mask, achieving a depth of nearly 300 nm. However, different crystal orientations exhibited significant differences in the etching sidewall angle. The −Z direction showed a favorable angle, reaching approximately 80°, while the +Z and −Y directions exhibited angles of only about 45° [4].

Focused-ion-beam etching encompasses direct-ion-beam cutting and ion-beam bombardment followed by wet etching, and it is characterized by its fast processing speed and excellent etching morphology. It is not affected by the crystal orientation of LNO, making it easy to achieve the desired etching sidewall angle [37,38]. However, due to its lower processing precision and significant surface damage to LNO, it is seldom used in the research of nanoscale LNO devices. By using high-energy ion implantation followed by wet chemical etching, the crystal structure of LNO is damaged, rendering the wet etching process non-isotropic. This leads to better results in terms of etching morphology. For instance, in 2013, Nicola et al. used high-energy Cu ion implantation followed by HF acid etching in LNO. The etching rate reached 100 nm/s, resulting in a smooth waveguide with a 90° sidewall angle and a depth of 2.75 μm [39].

In addition, there are combined dry- and wet-etching processes. For instance, the use of F-based gas etching combined with HF acid or SC-1 solution wet chemical etching to remove the etching by-products LiF has also resulted in favorable sidewall morphology [10,40]. In 2019, Osipov et al. employed SF_6_/O_2_ and SF_6_/Ar etching gases in ICP. They heated the LNO sample during etching and intermittently used hydrofluoric acid to corrode the LiF etching by-products, significantly increasing the etching rate of LNO and achieving excellent sidewall angles in etch depths at the micrometer-sized scale [41]. Another approach involves covering the LNO surface with metal Ti and performing high-temperature treatment, followed by electrochemical etching and subsequent wet chemical etching. This method has also been shown to be effective in processing LNO. In 2010, Zhang et al. covered the LNO surface with a Ti film and then annealed it in wet O_2_ at 1050–1100 °C. Afterward, they used a mixture of HF and HNO_3_ solutions for wet chemical etching. Despite a lower etching rate, they achieved an etch depth of approximately 260 nm [42].

Lastly, a clever process that has been widely studied in recent years is the dry etching/wet etching of proton-exchanged (PE) LNO. PE at temperatures above 180 °C can replace Li ions in the LNO surface with H ions (as shown in below chemical equation) [43]. This process effectively prevents the formation of LiF by-products during subsequent F-based gas etching, ultimately achieving a well-defined sidewall angle of 90°. For instance, in 2012, Deng et al. used PE LNO covered with a Cr mask and etched it using SF_6_/Ar gases to easily obtain an etching depth of over 3 μm, but the sidewall smoothness was relatively low [44].

LiNbO_3_ + *i*H^+^ ↔ H*_i_*Li_1−*i*_NbO_3_ + *i*Li^+^

Furthermore, PE LNO, due to its altered crystal structure, is more susceptible to wet chemical etching. This effectively circumvents the issue of different etching rates on different crystal orientations. For example, in 2020, Li et al. used proton-exchanged Z-cut LNO and wet-etched it with HF/HNO_3_, resulting in a symmetric etching morphology on both sides. The authors also conducted kinetic simulations of the PE reaction in LNO [36]. This clever etching process has received wide applications in LNO optical waveguide devices and surface acoustic wave devices. However, it cannot be applied to LNO domain-wall memory devices because PE damages the crystal structure, severely weakening its ferroelectric properties. This makes it impossible for LNO to undergo domain switching and domain-wall formation under voltage, ultimately leading to the failure of LNO domain-wall memory devices.

Different etching instruments have a significant impact on the etching morphology of LNO [33]. They can be mainly divided into two categories: RIE can only produce low-density plasmas, and ICP can generate high-density plasmas with the option of applying bias voltage. Moreover, the choice of etching masks also greatly affects the etching morphology and applications of LNO. Currently, the mainstream etching masks are made of metallic materials such as Cr, Ni, and Al [41,45,46]. Non-metallic masks like SiO_2_ and Si_3_N_4_, due to their relatively small etching selectivity with LNO, have received less attention in research. However, they have made a significant progress in studies involving Cl-based gas etching. The development of micro-nanostructures in LNO needs to be integrated with silicon-based integrated circuits. The use of metallic etching masks severely hampers its progress. Therefore, the challenge and focus lie in the use of non-metallic etching masks to achieve superior sidewall morphology in LNO.

In summary, achieving a well-defined etching sidewall angle without compromising the ferroelectric properties of LNO has become a challenging and critical issue, significantly impacting the development of LNO domain-wall memory chips. In response to these problems, this study investigated the effects of different etching machines capable of generating varying plasma densities, different etching masks (metal and non-metal), and various etching gases on LNO etching rates, selectivity, and sidewall morphology. Ultimately, using a metal mask resulted in excellent etching morphology, high selectivity, and favorable sidewall angles. When using a non-metal SiO_2_ mask, good etching morphology and a high selectivity were achieved. Finally, by combining dry etching, high-temperature metal corrosion, and wet etching, a novel process was developed to obtain a damage-free, perfectly smooth 90° inclined sidewall of LNO. These findings contribute to addressing the challenges associated with LNO domain-wall memory devices and provide potential strategies for improving the etching process to achieve desired sidewall characteristics without compromising the ferroelectric properties of LNO.

## 2. Experimental Methods

After undergoing ultrasonic cleaning using ethanol and acetone, 10 × 12 mm^2^ X-cut LNO single crystals with the 5 mol.% Mg dopant were used for the preparation of etching masks. The Cr and Ni metal films are commonly used etching masks for LNO. The fabrication processes for these masks are as follows: For the Cr masks, a 130 nm thick poly methyl methacrylate (PMMA) photoresist layer was spin-coated on the LNO surface, and then patterned using electron-beam lithography (EBL JEOL 6300FS, Tokyo, Japan). Cr films 50 nm thick were then grown using thermal resistance evaporator (Nano 36, Kurt J. Lesker, Pittsburgh, PA, USA) and were lift off. For the Ni masks, a 30 nm seed layer of Au was first prepared on the LNO substrate using magnetron sputtering (PVD-75, Kurt J. Lesker, Pittsburgh, PA, USA). Subsequently, a 130 nm PMMA photoresist layer was spin-coated, and then patterned using EBL. Finally, 60 nm thick Ni masks were fabricated using electroplating techniques and were lift off. Subsequently, the Au layer on the surface of LNO was removed using RIE with the etching parameters 40 sccm Ar, 2 Pa, 100 W, and 150 s. To achieve compatibility with conventional semiconductor processes, in addition to the mentioned metal masks, SiO_2_ masks, which are commonly used in semiconductor processes, were prepared on LNO. Unlike metal masks, the SiO_2_ masks were fabricated using etching. A 200 nm thick SiO_2_ layer was prepared using plasma-enhanced chemical vapor deposition (PECVD). Then, a 290 nm PMMA photoresist layer was spin-coated, and then patterned using EBL. Finally, the SiO_2_ etching masks were produced using RIE.

Sample 1 with Cr masks and sample 2 with Ni masks were etched using RIE (SAMCO Corporation, Kyoto, Japan). The process parameters were set as follows: 5 sccm SF_6_, 45 sccm Ar, 4 Pa, and 150 W.

Sample 3 with Cr masks was placed in molten benzoic acid liquid and subjected to proton exchange at 180 °C for 0.5 h. After removing the benzoic acid with ethanol, the sample underwent the same etching process as sample 1 in RIE.

Sample 4 with 50 nm SiO_2_ masks was subjected to a 2 h heat treatment at 400 °C in 10% H_2_ + Ar atmosphere, followed by 10 min wet etching in SC-1 solution at 85 °C.

Sample 5 with Cr masks underwent a 2 h heat treatment at 400 °C in 10% H_2_ + Ar atmosphere, and then the same etching process as sample 1 in RIE.

Sample 6 and sample 7 with Cr masks underwent the same etching process as sample 1 in RIE, followed by a 2 h annealing process at 400 °C in 10% H_2_ + Ar atmosphere (sample 7 had a 300 nm Al layer grown using PVD after etching and removed with a mixture of H_3_PO_4_:HNO_3_ (10:1) at 50 °C after annealing). Finally, both samples were subjected to a 10 min wet etching process using SC-1 solution at 85 °C.

Sample 8 with Cr masks and sample 9 with Ni masks were etched in ICP (Northern Microelectronics Corporation, Beijing, China). The process parameters were set as follows: 20 sccm SF_6_, 160 sccm Ar, 1.3 Pa, 800 W, with a bias power of 250 W.

Sample 10 with SiO_2_ masks was etched in ICP using the following recipe: 10 sccm SF_6_, 15 sccm BCl_3_, 50 sccm Ar, 1.3 Pa, 400 W, with a bias power of 250 W.

The detailed experimental processes of all samples are listed in Table 1.

The gratings dimensions of height and angle (*h* and *α*) were measured using field-emission scanning electron microscope images (SEM, Sigma HD, Carl Zeiss AG, Oberkochen, Germany). The X-ray diffraction pattern (XRD) was performed using a Cu K_α_ source (X’pert Pro3, Malvern PANalytical, Alemlo, Netherlands).

## 3. Results and Discussion

**Figure 1:**
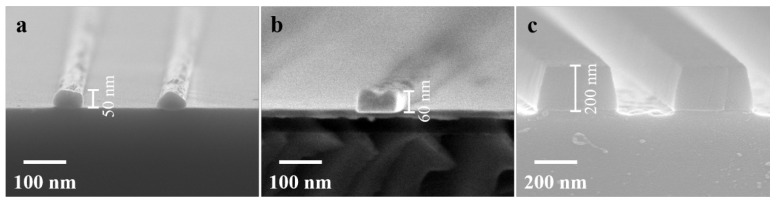
SEM images of (**a**) Cr masks, (**b**) Ni masks, (**c**) SiO_2_ masks.

SEM cross-sectional images of the Cr, Ni, and SiO_2_ masks are shown in Figure 1a–c, respectively. From the images, it can be observed that the evaporated Cr masks exhibit an elliptical shape at the top, the electroplated Ni masks appear to be rectangular, and the etched SiO_2_ masks have a trapezoidal shape. All three types of masks exhibit uniform and consistent characteristics, making them suitable for use as etching masks for LNO.

**Figure 2:**
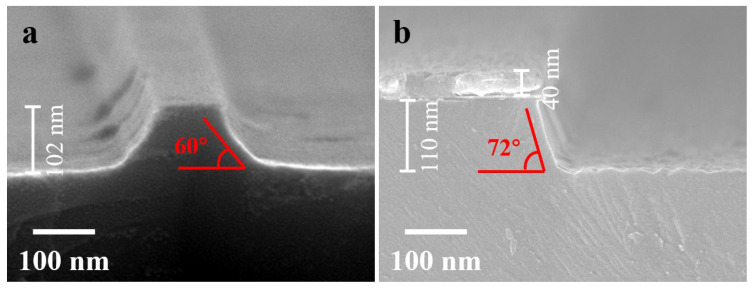
SEM images after RIE dry etching of (**a**) sample 1 with Cr masks and (**b**) sample 2 with Ni masks.

Figure 2a is SEM cross-sectional image of sample 1 with Cr masks after 10 min of RIE etching. It can be observed that the Cr mask is nearly completely consumed, and the sidewalls and bottom of the LNO grating are smooth. The etching depth is approximately 102 nm, corresponding to an etching rate of approximately 10 nm/min. The etching selectivity between the LNO and the mask is approximately 3:1. The sidewall inclination angle is approximately 60°. The conventional approach of the direct etching of LNO has been widely employed for fabricating various LNO devices, particularly emerging ferroelectric domain-wall memories. The reduced sidewall inclination has resulted in suboptimal retention performance and imprint effects in LNO memories [47,48,49,50]. Figure 2b shows the result of 10 min of RIE etching on sample 2 with a Ni mask. Approximately 40 nm of the Ni mask remains, and the depth of the LNO is approximately 110 nm. The etching rate for sample 2 is approximately 11 nm/min, resulting in an etching selectivity between the LNO and the mask of approximately 5.5:1. Compared to sample 1, sample 2 exhibits a significant improvement in the sidewall inclination angle, measuring approximately 72°. This improvement can be attributed to the superior morphology of the electroplated Ni mask compared to the evaporated Cr mask. The Ni mask has a higher hardness, which prevents the collapse of the grating sidewalls during the etching process, thus providing better protection for the LNO sidewalls [45,51].

It is evident that both types of masks do not yield sidewalls with angles close to 90°. This is due to the formation of a LiF byproduct during the etching process (As shown in the following chemical equation), which has a high melting point and is not easily vaporized. The LiF byproduct accumulates on the sidewalls of the LNO, forming sidewall protection and impeding further etching, resulting in smaller etching angles [30].

LiNbO_3_ + F* → LiF↓ + NbF_x_↑ + O_2_↑ + OF_2_↑

**Figure 3:**
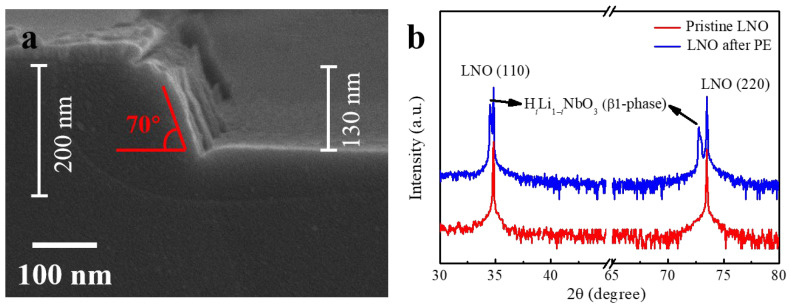
(**a**) SEM image of sample 3 after dry etching in RIE following proton exchange. (**b**) XRD patterns of LNO before and after proton exchange.

To address the issue of LiF by-product accumulation during the etching process, sample 3 with Cr masks underwent PE and subsequent dry etching in RIE for 10 min. The SEM cross-sectional image of sample 3 is presented in Figure 3a. It is evident that sample 3 exhibited an etching depth of approximately 130 nm, an etching rate of around 13 nm/min, and an etching angle of about 70°. In comparison to sample 1, which underwent direct dry etching, sample 3 subjected to PE showcased an increased etching rate and a notable enhancement in the inclination angle, albeit with a slightly rougher sidewall surface. Upon close examination, it can be observed that after PE in molten benzoic acid at 180 °C for 0.5 h, a distinct boundary existed between the PE-LNO and the intrinsic LNO, with an exchange depth of approximately 210 nm. The XRD characterization, shown in Figure 3b, of both non-PE and PE LNO reveals distinct phase transition peaks near the (110) and (220) LNO substrate peaks. This signifies a discrepancy in the lattice spacing between the PE-H*_i_*Li_1−*i*_NbO_3_ and the substrate LNO [36]. The observed phase transition is primarily attributed to an increase in lattice constants and lattice distortion of the H*_i_*Li_1−*i*_NbO_3_. According to previous studies, this phase is identified as the β1 phase of LNO. With longer PE durations and higher temperatures, deeper exchange layers along with the emergence of new β2 and β3 phases can be generated [52,53,54]. Additionally, these phase transitions introduce more defects, contributing to the accelerated etching rate and increased sidewall roughness in PE-LNO.

**Figure 4:**
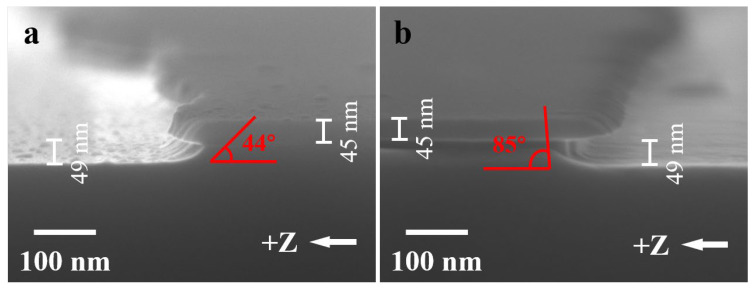
SEM images for (**a**) +Z direction and (**b**) −Z direction of sample 4 without the Al thin-film coverage, after annealing in a reducing atmosphere and wet etching with SC-1 solution.

Under a high-temperature reducing atmosphere, Li on the surface of LNO will volatilize, and the oxygen vacancy concentration on the surface will also increase [55]. This phenomenon prompts the formation of voids on the LNO surface, rendering its more easy etching in both dry and wet etching processes. This perspective holds promise as a potential avenue for novel approaches in the etching of LNO. The SEM images in Figure 4a,b depict LNO with SiO_2_ masks, subjected to treatment in a 10% H_2_ + Ar atmosphere at 400 °C, followed by wet etching in SC-1 solution at 85 °C for 10 min. As demonstrated in the figures, the etching depth of LNO was approximately 49 nm, and the upper surface of LNO also exhibited lateral erosion in a horizontal direction, resulting in lateral separation from the overlying SiO_2_ masks. However, distinctive etching rates can be observed for LNO in the +Z and −Z directions, leading to dissimilar sidewall inclinations of 44° and 85°, respectively. Moreover, the lateral erosion distances differed between these directions, with the +Z direction notably smaller than the −Z direction.

**Figure 5:**
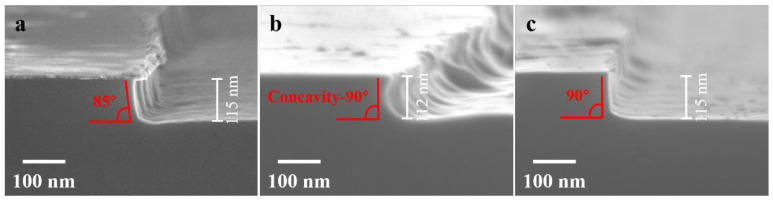
(**a**) SEM image of sample 5 without the Al thin-film coverage, after annealing in a reducing atmosphere and dry etching with RIE. (**b**) SEM image of sample 6, after dry etching with RIE and annealing in a reducing atmosphere without the Al thin film coverage, followed by wet etching with SC-1 solution. (**c**) SEM image of sample 7, after dry etching with RIE and annealing in a reducing atmosphere with the Al thin-film coverage, followed by wet etching with SC-1 solution.

Sample 5 with Cr masks underwent a similar process to sample 4. It was subjected to treatment in high-temperature reducing conditions followed by a 10 min dry-etching step in RIE. The SEM image in Figure 5a shows the post-etching result. The etched depth of the LNO is approximately 115 nm, corresponding to an etching rate of about 11.5 nm/min, and displaying an etching sidewall angle of around 85°. In comparison with the direct dry etching of sample 1, the high-temperature reduction treatment and etching process applied to sample 5 caused a slight increase in the etching rate but a significant enhancement in the sidewall angle.

On the contrary, sample 6 with Cr masks, was subjected to a reversed procedure. It first underwent a 10 min dry-etching step in RIE, followed by treatment in high-temperature reducing conditions and subsequent 10 min wet etching in SC-1 solution. The SEM image in Figure 5b reveals a final depth of around 112 nm for the Cr mask-removed LNO. Notably, an inward concavity shape is observed on the sidewall. Interestingly, the LNO grating’s top part in sample 6 did not exhibit lateral etching, while the mid-section of the sidewall showed lateral etching. This phenomenon raises suspicions that the top Cr mask might have diffused into the underlying LNO at elevated temperatures, leading to alloy formation [56,57,58]. The alloyed LNO layer at the top appeared less susceptible to wet etching than the middle part, resulting in the formation of the inward concavity sidewall. While the inward concavity sidewall is not favorable for LNO device fabrication, it does, however, offer new avenues for the refinement of etching techniques.

Subsequently, sample 7 with Cr masks underwent a process nearly identical to sample 6, except for an additional step involving the deposition of a 300 nm Al layer through PVD after the dry etching of LNO. Prior research has indicated that LNO covered by highly active metal films, when annealed under reducing atmospheres, can lead to a significant reduction of oxygen in the LNO due to the metal’s strong reactivity at elevated temperatures [55]. This ultimately results in the creation of a porous and loosely packed surface on the LNO, rendering it more susceptible to dry and wet etching. As depicted in Figure 5c, the LNO achieved a final depth of approximately 115 nm, and the sidewall exhibited a pretty 90° angle. In the case of LNO subjected to dry etching, followed by the high-temperature annealing of the deposited Al layer, and then wet etching, the presence of the Cr-LNO alloyed layer on top rendered it less susceptible to wet etching. Consequently, the sidewall, initially at a 60° angle, was enhanced into a 90° angle. This hybrid process ingeniously uses the alloyed LNO layer, resulting in smooth sidewalls with precise 90° angles. Moreover, this process preserves the ferroelectric properties of LNO, making it suitable for large-scale fabrication of LNO domain-wall memory arrays.

**Figure 6:**
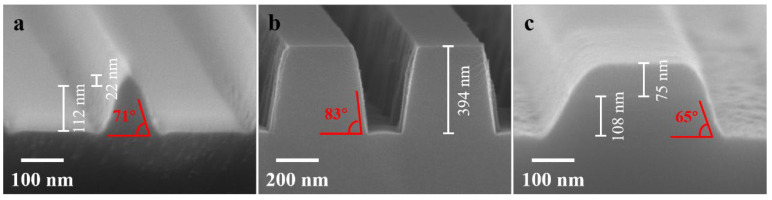
SEM images after ICP dry etching of (**a**) sample 8 with Cr masks, (**b**) sample 9 with Ni masks and (**c**) sample 10 with SiO_2_ masks.

The SEM image of sample 8 with Cr masks, after 3 min of ICP etching, is shown in Figure 6a. The etched depth of the LNO is approximately 112 nm, corresponding to an etching rate of approximately 37 nm/min. The sidewall inclination angle is approximately 71°. However, the grating top width is near to 0, indicating that the Cr mask was no longer effective as a mask. This is due to the tendency of the Cr mask to undergo sidewall indentation during the etching process, resulting in a triangular shape for the overall grating. Due to the higher plasma density generated by the ICP instrument compared to RIE, although it increased the etching rate of LNO and correspondingly improved the etching angle to some extent, it also resulted in significant damage to the Cr mask.

Figure 6b displays the result of 5 min of ICP etching on sample 9 with a Ni mask, where the Ni mask and Au seed layer were removed. The depth of the LNO is approximately 394 nm, with an etching rate of approximately 79 nm/min, and the etching inclination angle is approximately 83°. Compared to the Cr mask, due to the greater hardness of Ni, it exhibited better shape retention during ICP etching, consequently resulting in a higher inclination angle of LNO sidewalls. Additionally, the etching rate of LNO in sample 9 was relatively higher compared to that observed with the Cr mask. This is because the F ions participate more in the reaction with Cr than with Ni during the high-density plasma-etching process [41,45]. As a result, when using a Cr mask, the consumption of etching gas is higher than when using a Ni mask, leading to a slight difference in the etching rate of LNO.

Finally, to etch LNO gratings to a certain depth using SiO_2_ masks, the same etching recipe as that used for metal masks cannot be applied. This is because F-based gases etch SiO_2_ at a significantly higher rate [29,34]. Therefore, in the case of sample 10 with SiO_2_ masks, the proportion of F-based gases in the recipe was reduced while increasing the proportion of Cl-based gases, which exhibited lower etching rates for SiO_2_. Figure 6c illustrates the result of 1 min of ICP etching on sample 10. The remaining thickness of SiO_2_ is approximately 75 nm, while the etched depth of LNO is approximately 108 nm, resulting in an etching rate of 108 nm/min and an etching selectivity of 0.86:1. Like the Cr masks, the SiO_2_ masks also experienced sidewall indentation during the etching process, leading to a degradation of the etching inclination angle of LNO to approximately 65°. Although the etching inclination angle of LNO was relatively poor compared to previous studies that employed non-metal masks for etching LNO, a significant improvement in etching selectivity was achieved [35]. Further research holds the potential to achieve even better etching profiles, thereby facilitating the effective integration of LNO memories with traditional semiconductor processes in the future.

Table 1 compiles the key etching processes for the 10 samples and presents essential parameters including the etching rate, etching selectivity ratio, and sidewall angle. The high-hardness Ni masks demonstrate excellent mask integrity in both RIE and ICP etching processes for LNO. Moreover, they yield superior etching angles and slightly higher etching rates compared to the Cr masks. Employing a post-dry-etching annealing process with Al metal-film coverage at an elevated temperature in a reducing atmosphere enhances the sidewall profile from an initial 60° to a perfect 90°. Finally, when utilizing SiO_2_ masks and ICP with a recipe comprising Cl-based, F-based, and Ar gases, remarkable etching rates and favorable etching selectivity are achieved. However, the sidewall inclination of 65° still requires improvement.

## 4. Conclusions

In conclusion, this research underscores the significance of understanding and optimizing etching techniques for LNO nanofabrication. The outcomes of this study hold potential for advancing the integration of LNO into various applications, thereby contributing to the advancement of cutting-edge technologies across multiple domains. This study delved into the field of LNO etching techniques, which play a pivotal role in enabling the transformation of its unique properties into functional devices. Building upon existing etching research, this investigation explored diverse etching strategies, capitalizing on instruments capable of generating varied plasma densities. The utilization of RIE dry etching, proton exchange-enhanced etching, wet etching following high-temperature reduction treatment, and hybrid dry and wet etching unveiled new avenues for LNO nanofabrication. The optimized approach, combining RIE dry etching with subsequent wet etching after high-temperature reduction treatment, yielded an impressive etching rate of 10 nm/min and pristine 90° sidewall angles. Furthermore, by embracing ICP dry etching, significantly enhanced etching rates of 79 nm/min alongside steep sidewall angles of 83° were achieved. Additionally, the integration of SiO_2_ masks facilitated a high etching rate of 108 nm/min, coupled with a respectable etching selectivity ratio of 0.86:1. The diverse range of etching conditions generated exceptional results, paving the way for multiple pathways in harnessing the versatile capabilities of LNO.

## Figures and Tables

**Table 1:** Etching processes and results for the 10 samples: etching rates, sidewall angles, and etching selectivity.

Samples	Masks	Equipment	Processes	Etching Rates (nm/min)	Sidewall Angles (°)	Etching Selectivity Ratio
1	Cr	RIE	Dry etching	10	60	~3:1
2	Ni	RIE	Dry etching	11	72	~5.5:1
3	Cr	RIE	Proton exchange, then dry etching	13	70	~4:1
4	SiO_2_	/	High temperature treated in reduction atmosphere, then SC-1 wet etching	4.9	44–85	/
5	Cr	RIE	High temperature treated in reduction atmosphere, then dry etching	11.5	85	~3.5:1
6	Cr	RIE	Dry etching, then high temperature treated in reduction atmosphere, then SC-1 wet etching	10	Concavity-90	~3:1
7	Cr	RIE	Dry etching, then high temperature treated with Al in reduction atmosphere, then SC-1 wet etching	10	90	~3:1
8	Cr	ICP	Dry etching	37	71	~4:1
9	Ni	ICP	Dry etching	79	83	~6:1
10	SiO_2_	ICP	Cl-based gases dry etching	108	65	0.86:1

## Data Availability

The data reported in this manuscript are available on request from the corresponding author.
